# Improved Classification of White Blood Cells with the Generative Adversarial Network and Deep Convolutional Neural Network

**DOI:** 10.1155/2020/6490479

**Published:** 2020-07-09

**Authors:** Khaled Almezhghwi, Sertan Serte

**Affiliations:** Electrical and Electronic Engineering, Near East University, Nicosia, North Cyprus, Mersin 10, Turkey

## Abstract

White blood cells (leukocytes) are a very important component of the blood that forms the immune system, which is responsible for fighting foreign elements. The five types of white blood cells include *neutrophils*, *eosinophils*, *lymphocytes*, *monocytes*, and *basophils*, where each type constitutes a different proportion and performs specific functions. Being able to classify and, therefore, count these different constituents is critical for assessing the health of patients and infection risks. Generally, laboratory experiments are used for determining the type of a white blood cell. The staining process and manual evaluation of acquired images under the microscope are tedious and subject to human errors. Moreover, a major challenge is the unavailability of training data that cover the morphological variations of white blood cells so that trained classifiers can generalize well. As such, this paper investigates image transformation operations and generative adversarial networks (GAN) for data augmentation and state-of-the-art deep neural networks (i.e., VGG-16, ResNet, and DenseNet) for the classification of white blood cells into the five types. Furthermore, we explore initializing the DNNs' weights randomly or using weights pretrained on the CIFAR-100 dataset. In contrast to other works that require advanced image preprocessing and manual feature extraction before classification, our method works directly with the acquired images. The results of extensive experiments show that the proposed method can successfully classify white blood cells. The best DNN model, DenseNet-169, yields a validation accuracy of 98.8%. Particularly, we find that the proposed approach outperforms other methods that rely on sophisticated image processing and manual feature engineering.

## 1. Introduction

Blood is vital for life, and many functionalities of the body organs rely on healthy blood. The healthiness of blood can be assessed by analysing the blood constituents (i.e., cells). Generally, the blood contains cells and a liquid portion known as the plasma [1]. The blood cells constitute about 45% of the blood volume, while the plasma constitutes the remaining 55% [2, 3]. The blood cells are of three types that include the *red blood cells* (*erythrocytes*), *white blood cells* (*leukocytes*), and *Platelets* (*thrombocytes*) [4]. The red blood cells make up 40–45% of the blood, while the white blood cells make up about 1% of the blood [3, 5, 6]. The three different blood cells have different functions for the body organs. However, the white blood cells are produced in the bone marrow and are a very important constituent of the blood. White blood cells are primarily responsible for the body's immune system that serves as a defence mechanism against foreign elements in the body, especially disease-causing elements.

**Figure 1:**
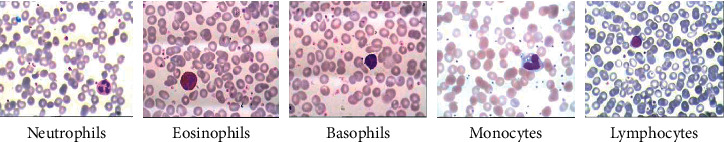
The five types of white blood cells.

**Figure 2:**
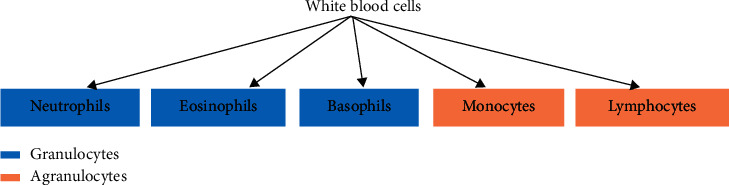
Classification of blood cells.

**Figure 3:**
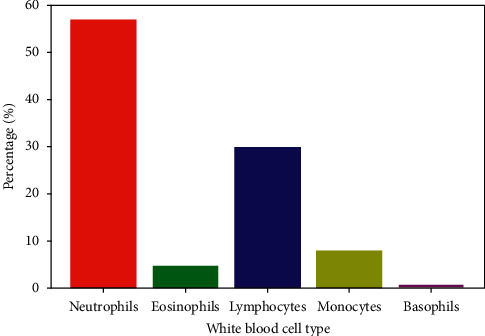
Proportion of the five different types of white blood cells in the blood [5].

White blood cells are of five different types, which include neutrophils, eosinophils, lymphocytes, monocytes, and basophils; see Figure 1. These blood cells can be further divided into two broad groups, granulocytes and agranulocytes (nongranulocytes) [7]; see Figure 2. Granulocytes are the white blood cell types that possess visible granules, while agranulocytes are the types with no visible granules when observed under a microscope [7]. Neutrophils, eosinophils, and basophils belong to the granulocytes class, while monocytes and lymphocytes belong to the agranulocytes class. We note that the percentages of neutrophils, eosinophils, lymphocytes, monocytes. and basophils are 40–60%, 1–4%, 20–40%, 2–8%, and 0.5–1% in the blood, respectively [5]; see Figure 3. The five types of white blood cells have different functionalities and reflect different conditions about the health of patients (subjects). As such, identifying the different white blood cells is often of interest. Particularly, correct identification results in the possibility of counting the different white blood cells to assess their presence in the correct or expected proportions. Furthermore, different white blood cells upon identification can be isolated for detailed examination for abnormalities. The quantitative and qualitative examination of white blood cells reveal a lot about the health of patients. For example, it is possible to assess patients for health conditions including leukaemia, immune system disorders, and cancerous cells [8]. Conventionally, the identification requires a laboratory setting where acquired images of blood cells are stained using special chemicals (i.e., reagents) and, afterwards, examined under a microscope by a specialist. However, this process is delicate and requires that there is no (or minimal) examination error by the human specialist. Unfortunately, specialists can often become fatigued after several hours of examination and make inaccurate identification of the different white blood cells.

This paper investigates the automatic classification of white blood cells using data augmentation techniques and DNNs that are fast, accurate, and cost-effective as an alternative approach to the laboratory setting. The data augmentation techniques employed are image transformation operations and GAN image generation. Namely, we explore the state-of-art DNNs such as VGG [9], ResNet [10], and DenseNet [11] that are pretrained on the CIFAR-100 dataset [12] for classifying white blood cells into one of the following: *neutrophils*, *eosinophils*, *lymphocytes*, *monocytes*, or *basophils*.

A major advantage over existing methods is that our proposal requires no specialized image preprocessing and feature engineering for robust classification. Our main contributions in this paper are as follows.

1. Propose DNNs that are trainable end-to-end for the automatic classification of white blood cells into the five different types of white blood cells, which include neutrophils, eosinophils, lymphocytes, monocytes, or basophils.
2. Explore several DNN architectures, including those initialized using pretrained weights to boost classification performance on such an important medical task.
3. Investigate data augmentation techniques such as transformation operations and GAN-generated instances to further improve the classification performance of the DNNs.
4. Demonstrate that the proposed system directly works well with acquired images and outperforms the methods that employ painstaking image preprocessing and feature engineering. The experimental results reported reflect the state-of-the-art results.

The remaining sections in this paper are divided as follows. Related works are discussed in Section 2.  Section 3 presents the proposed framework for the classification of white blood cells. Extensive experiments using different model architectures and training settings, along with the discussion of results, are given in Section 4. The method and findings in this paper are summarized as the conclusion in Section 5.

## 2. Related Works

The classification of blood cells has been a subject of interest in the last few decades. This interest seems to have been considerably influenced by the general growth of machine and deep learning for unconventional tasks such as classifying chest X-rays [13–15], red blood cell [16, 17], segmenting medical images [18–21], breast cancer determination [22, 23], and Alzheimer's disease [24, 25]. For instance, the work [26] proposed the identification of the red blood cell, white blood cell, and platelet using the popular YOLO object detection algorithm and deep neural networks for classification with interesting results.

The automatic classification of blood cells is commonly achieved using advanced image preprocessing and feature extraction. In [27], image preprocessing techniques such as contrast stretching, opening, edge detection, dilation, filling, cropping, and minimum intensity homogenization were applied as preprocessing steps for images of white blood cells.

Subsequently, the work [27] extracted features including the area, perimeter, convex area, solidity, major axis length, orientation, filled area, eccentricity, rectangularity, circularity, the number of lobes, and mean gray-level intensity of the cytoplasm; a total of 23 features were extracted for describing the different cells. Afterwards, feature selection was carried out to reduce the number of extracted features from 23 to 3. Finally, classifiers such as k-nearest neighbours, a feedforward neural network, a radial basis function neural network, and a parallel ensemble of feed forward neural networks were trained for discriminating the different white blood cell types. In another work [28], the acquired grayscale images of white blood cells are preprocessed using median filtering, cell localization via thresholding operations, and edge detection. From the preprocessed white blood cells, 10 different features were extracted for training, which resulted in a classification accuracy of about 90%. The work [29] proposed the classification of white blood cells including lymphocytes, monocytes, and neutrophils; eosinophils and basophils were not considered. Again, [29] relied on image preprocessing such as grayscale conversion, histogram equalization, erosion, reconstruction, and dilation. The resulting images were segmented via thresholding operations. Finally, classification was performed using 5 or 6 different features extracted from the segmented images. Although good results were reported, the number of test samples was extremely small. There were 34, 12, and 29 test samples for lymphocytes, monocytes, and neutrophils, respectively.

In [7], the acquired digitized scans of white blood cells were segmented using the active contour technique. Some features were extracted from the segmented images and then classified using the Naïve Bayes model with Laplacian correction. The work [31] employed k-means clustering for segmenting white blood cells from the acquired images and performed feature extraction, feature selection via Principal Component Analysis (PCA), and classification using an artificial neural network. In [32], the Fast Relevance Vector Machine (F-RVM) was proposed for the segmentation and classification of white blood cells. They posit that F-RVM is easier to train and requires a small time for inference than the Extreme Learning Machine (ELM) and standard RVM. Otsu's thresholding method was used in [30] for segmenting white blood cells, after which mathematical morphological operations were applied to eliminate all elements that have no resemblance with white blood cells. Following the segmentation results, features were extracted from the cell nucleus for training a Naïve Bayes classifier. Although promising results were reported in the aforementioned related works, a major problem is the extremely small size of the dataset used for training and testing. Many of the works relied on 20–40 images per class for training and testing the proposed models. In real-life, the diversity of the acquired images of white blood cells can render models trained on small datasets ineffective.

The comparison of the approach proposed in this paper with earlier works is summarized in Table 1.

## 3. Proposed Classification of White Blood Cells

**Figure 4:**
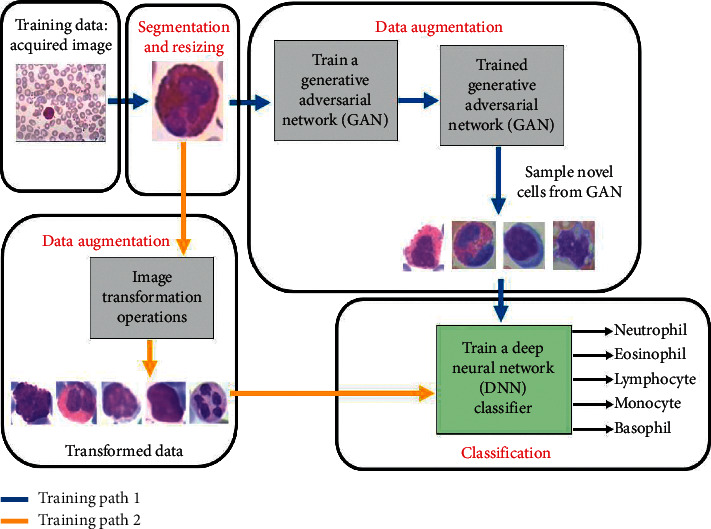
Proposed framework for the improved classification of the white blood cell type. Training path 1: GAN data augmentation flow for training a DNN classifier. Training path 2: transformation operations data augmentation flow for training a DNN classifier.

In this section, we present the proposed framework for the classification of white blood cells into the five different classes. The proposed framework is shown in Figure 4. The main components of the proposed system include (i) white blood cell segmentation and resizing, (ii) the data augmentation process via transformation operations or GAN generation, and (iii) DNN training. These components are discussed in succession as follows.

### 3.1. White Blood Cell Segmentation

The LISC blood cells dataset [33] is used in this paper. The original images contain white blood cells along with other background elements that are irrelevant for classifying the different types of white blood cells. The irrelevant background elements occupy a large portion of the images (i.e., Figure 1), and thus, the images for training the DNN classifiers have low signal to noise ratios that can negatively affect the classification performance.

**Figure 5:**
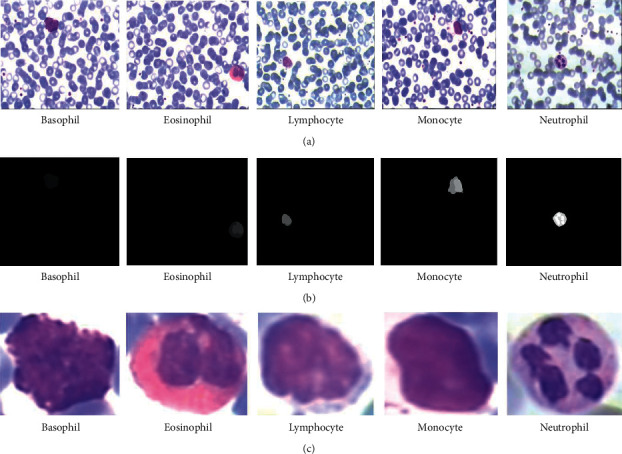
Segmentation of white blood cells. (a): original images of different blood cells. (b): segmentation masks for the white blood cells. (c): segmented white blood cells from the original images.

Consequently, we segment the portion of the images containing the white blood cells using the masks given in the dataset; the bounding box coordinates that capture the nonzero pixels in the given masks are used to crop out (i.e., segment) the white blood cells in the images. Lastly, the segmented white blood cells are resized to fit as the input of the constructed DNN models. Samples of the white blood cells and their corresponding masks are shown in Figure 5.

### 3.2. Data Augmentation to Improve the DNN Classification Performance

A major challenge for developing accurate classification systems for white blood cells is insufficient data for training; data instances that cover the morphological variations of the different cells are usually unavailable. Small number of data instances from a class typically creates class imbalance that biases learning; models learned from imbalanced data typically perform poorly during testing [34]. The following sections discuss the different approaches that are explored for generating additional data, which can be used to improve the classification accuracy of the DNN classifiers.

#### 3.2.1. Additional Data via Data Transformation Operations

Herein, image transformation operations are employed for generating additional data instances from the original data. Specifically, the image transformation operations applied include random rotations in the angle range of 0–360°, random shearing in the angle range of 0–20° counterclockwise, random horizontal flips, and random height and width shift of up to 20% of the image height and width. The aforementioned transformation operations are applied to generate the desired number of data instances.

#### 3.2.2. Additional Data Using the Generative Adversarial Network (GAN)

**Figure 6:**
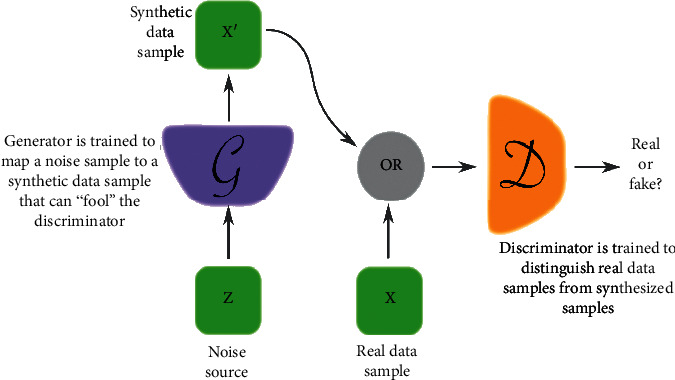
Generative adversarial network (GAN) operation [36].

The GAN is a generative model that can be used to generate novel data points from a distribution that is similar to the training data. The GAN is essentially based on the min-max game theory [35], where the discriminator and generator work in opposition to outperform each other. The generator is tasked to generate fake (i.e., synthetic) novel data instances that look real, while the discriminator works to identify the fake instances; see Figure 6. The detailed operation and training objective of the GAN are in [35, 36].

The aim is that the generator via this game learns to generate data instances that are similar to the real data instances. As such, we propose to generate novel data points by training a GAN on the original data. The data points generated from the trained GAN are different instances of the original data instances and can indeed contribute to learning features that generalize to unseen data instances during testing. Specifically, we consider the conventional GAN [35] for generating novel data points as addition data. The training details of the GAN are given in Section 4.2.1.

#### 3.2.3. Additional Data Using Both Data Transformation Operations and a Trained GAN

For this approach of generating additional data for training, the data instances obtained from transformation operations are combined with the novel instances generated from the trained GAN. These new data are then used for training the different DNN models. Specifically, we are interested in observing if such data combination can improve the performance of the trained DNN models.

### 3.3. Deep Neural Networks for White Blood Cells Classification

**Figure 7:**
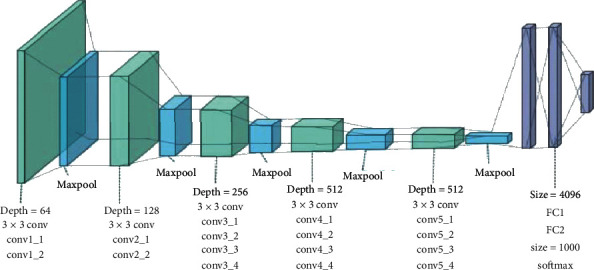
VGG-19 model architecture [37].

**Figure 8:**
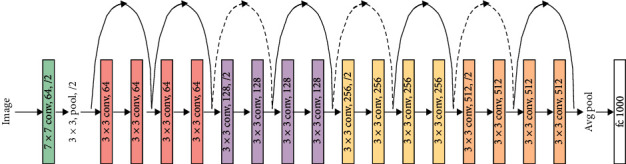
ResNet-18 model architecture [10].

**Figure 9:**
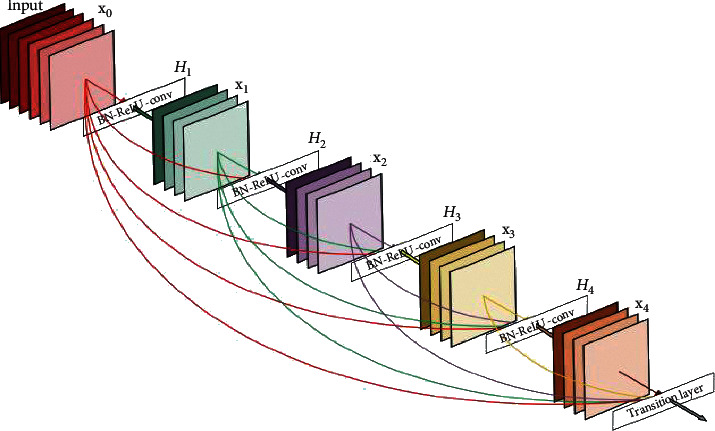
DenseNet model architecture [11].

For the classifier, different state-of-the-art DNNs including VGG, ResNet, and DenseNet are trained on the prepared datasets. Figures 7–9 show the basic model architecture of the VGG-19, ResNet-18, and DenseNet, respectively. Note that the actual number of layers in the different models can vary. The VGG model uses a single path for information flow from the input layer to the output layer. The ResNet uses skip connections that permits the additional of the outputs from lower layers to the outputs of higher layers to improve model training; see [10] for details on the operation of the ResNet. The DenseNet employs skip connections that permit the concatenation of the outputs of lower layers to the outputs of higher layers. For the DenseNet, the output of every layer is concatenated to the outputs of all the preceding layers in the model; the detailed operation of the DenseNet is in [11]. Furthermore, we consider three major training settings that can impact the performance of the DNNs, especially in the absence of abundant training data. These settings are discussed as follows.

#### 3.3.1. Random Initialization of the DNN

The DNN weights are initialized randomly and trained from scratch using popular initialization schemes such as [38, 39]. For random initialization, the objective is to break the symmetry in the weights space at the start of training such that the DNN can explore various parts of the solution space. That is, random initialization discourages the DNN optimization from being stuck in a particular basin of attraction, which may be quite suboptimal in the solution space.

#### 3.3.2. DNN Weights Initialization from Weights Trained on a Large Dataset

DNN weights are initialized from the weights trained on the CIFAR-100 classification dataset, which contain 50,000 natural training images that belong to 100 different classes [12]. Initializing the weights of DNNs from weights trained on large datasets has been shown to improve model generalization, especially when the available training data are not abundant [40, 41]. The main concept behind this success is that DNNs typically contain several millions of parameters and, thus, have the propensity to overfit in the absence of large training data.

Interestingly, it is known that the weights in the early layers of DNNs trained on very large datasets resemble generic features and hence, can be employed for feature extraction in other tasks [42]. Generally, after initializing the DNN using the weights trained on the CIFAR-100 dataset, the specific layer weights to be updated (i.e., trained) using the current dataset are heuristically determined via experiments; this process is termed “fine-tuning” [43]. Common approaches for fine-tuning DNNs are (i) updating the weights of all layers and (ii) updating the weights of specific layers and freezing (fixing) the weights of other layers. The weights of the softmax (i.e., output) layer is usually initialized randomly and trained from scratch. By experimenting with the different aforementioned methods of initializing the weights of the DNNs, we can observe the advantage of one method over the other based on the performance.

#### 3.3.3. Deep Convolutional Neural Network Depth

The depth (i.e., the number of parameterized layers) of DNNs is a critical factor for their performance [44]; deeper DNNs usually generalize better than shallow ones [10, 44, 45]. As such, given the aforementioned DNNs that are considered in this paper, we observe the impact of depth on their performance for the classification of the different types of white blood cells. For the VGG model, architectures with 16 and 19 layers are considered; for the ResNet, architectures with 18 and 50 layers are considered; for the DenseNet, architectures with 121 and 169 layers are considered.

## 4. Experiments

In this section, the details of the dataset and experiments performed are presented, along with the specific settings, results and discussion. All experiments are performed using a workstation with a 32 GB of Random Access Memory (RAM), an Intel core-i7 processor, Nvidia GTX1080Ti GPU (Graphics Processing Unit), and running Windows 10 operating system. All implementations employ the Keras deep learning framework with Tensorflow backend.

### 4.1. Original Dataset

For demonstrating that the proposed framework improves the classification of white blood cells, we use the LISC dataset [33], which covers all the five different types of white blood cells. Altogether, the dataset has 242 data instances. The number of data instances per class in the original dataset is given in Table 2.

### 4.2. Training Settings for Models

This section presents the details of the different training settings and data augmentation schemes. For all the given tables, “instances” is abbreviated as “inst.” for brevity.

#### 4.2.1. GAN Training Settings

The data given in Table 2 are used to train a GAN with two convolutional layers and one fully connected layer for both the generative and discriminative networks. Following the work [35], the GAN is trained for 60 epochs using a learning rate of 0.01 and a momentum rate of 0.5.

#### 4.2.2. DNN Classifier Training and Evaluation Settings

The different DNNs are trained using the minibatch gradient descent method. A batch size of 128 is used for all the models. All the DNN models with randomly initialized weights are trained using an initial learning rate of 0.1 for 300 epochs. All the DNN models initialized using the weights trained on the CIFAR-100 dataset [12] are trained using an initial learning rate of 0.005 for 150 epochs. A momentum rate of 0.9 is used for all models, and the initial learning rate is reduced by a factor of 0.1 every time the training loss did not reduce by 0.001 for 5 consecutive epochs. A weight decay value of 1 × 10^−4^ is used for regularizing all the DNN models. The segmented white blood cells images are resized to 32 × 32 pixels for input to all the DNN models.

For evaluating the performance of the trained DNNs, we employ a 10-fold cross-validation scheme, given the size of the dataset. Essentially, we partition the data into 10 segments, train the DNN models on 9 different data folds, and validate on the remaining data fold. This process is repeated 10 times using different 9 data folds for training and 1 different data fold for testing. The average validation accuracy over the 10 different data folds is reported.

### 4.3. Data Augmentation Methods

#### 4.3.1. Transformation Operations for Data Augmentation

Herein, we apply the aforementioned data transformation operations given in Section 3.2.1 to the data instances in the different classes to augment the original dataset. We generate three new datasets referred to as Trans_aug1, Trans_aug2, and Trans_aug3 that now have 100 data instances/class, 150 data instances/class, and 200 data instances/class, respectively. Each of the aforementioned different datasets is used to train and validate the different DNN models.

#### 4.3.2. GAN Method for Data Augmentation

**Figure 10:**
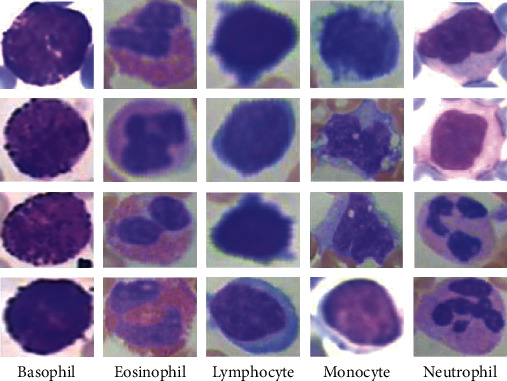
Samples of data instances generated from the trained GAN for data augmentation.

From the trained GAN in Section 3.2.2 and Section 4.2.1, we generate three different datasets referred to as GAN_aug1, GAN_aug2, and GAN_aug3 that have 100 data instances/class, 150 data instances/class, and 200 data instances/class, respectively. Some of the data instances generated from the trained GAN are shown in Figure 10.

### 4.4. Results and Discussion

The results of the DNN models trained and tested on segmented white blood cells are in given in Tables 3–10.  Table 3 shows the results of the DNNs trained on the original data (i.e., without data augmentation) using randomly initialized weights.  Table 4 shows results similar to Table 3, except that the DNN weights were pretrained on the CIFAR-100 dataset.  Table 5 shows the results of the DNN models that were initialized randomly and trained using Trans_aug1, Trans_aug2, and Trans_aug3.

Table 6 reports the results of the DNN models that were initialized with the pretrained weights using Trans_aug1, Trans_aug2, and Trans_aug3 datasets. In Table 7, the results of the DNN models initialized with random weights and trained using GAN_aug1, GAN_aug2, and GAN_aug3 datasets are given.  Table 8 gives the results of the DNN models trained with pretrained weights on GAN_aug1, GAN_aug2, and GAN_aug3 datasets.

We perform additional experiments by combining the data instances obtained from translation operations and the trained GAN. As such, we obtain three different datasets referred to as Trans_aug1 + GAN_aug1, Trans_aug2 + GAN_aug2, and Trans_aug3 + GAN_aug3 that have 200 data instances/class, 400 data instances/class, and 600 data instances/class, respectively. In Table 9, the results of the DNN models initialized with random weights on Trans_aug1 + GAN_aug1, Trans_aug2 + GAN_aug2, and Trans_aug3 + GAN_aug3 datasets are reported. The results of the DNN models initialized with the pretrained weights and trained on Trans_aug1 + GAN_aug1, Trans_aug2 + GAN_aug2, and Trans_aug3 + GAN_aug3 are given in Table 10. The overall observations based on experimental results are as follows.

We observe that the DNN models that employed pretrained weights consistently outperform the same DNN models trained on a similar dataset, but with randomly initialized weights.

It is seen from Tables 3to 10 that the ResNet and DenseNet models, which have several parameterized layers and use skip connections, outperform the VGG models. Furthermore, it is observed that data augmentation improves the performance of all the models; compare Table 3 with Tables 4to 10. Specifically, using similar number of data instances/class, the augmented datasets obtained from the trained GAN lead to better DNN performances as compared to the augmented datasets obtained from image transformation operations. Interestingly, combining the data instances obtained from the trained GAN with the data instances obtained from the image transformation operations results in further improvement in results as compared to using the augmented data obtained from either the trained GAN or the image transformation operations.

**Figure 11:**
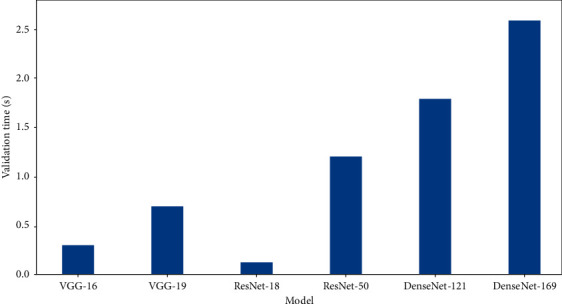
Time for the DNN models to perform inference on the validation data. The original data without data augmentation given in Table 2 is used for this experiment.

From the computational perspective, Figure 11 shows the time required by the different DNN models to perform inference using the validation data from the 10-fold cross-validation training scheme. It is seen that the best models, ResNet-50, DenseNet-121, and DenseNet-169, incur the largest inference times. This is not surprising, given that they have several parameterized layers and, thus, require more time for computing their final outputs.

Table 11 reports the results comparison with earlier works; the best results in this paper are given in bold. Particularly, we consider, for comparison, earlier works that perform the classification of the 5 different types of white blood cells. We note that the DNN models proposed in this paper outperform the models from earlier works, which employed 10-fold CV.

## 5. Conclusions

The analysis of the constituents of the white blood cells of patients can reflect their health conditions. The different constituents are normally present in different proportions and play different roles for the well-being of patients. However, the laboratory preparation and manual inspection of microscopic images of white blood cells can be too delicate and erroneous. Subsequently, inaccurate assessment of patients' conditions can occur. In using machine learning models for classification, insufficient training data to cover the morphological variations of the different white blood cells is a major challenge. As such, this paper investigates data augmentation techniques and the deep neural network for the automatic classification of white blood cells into the five types that include neutrophils, eosinophils, lymphocytes, monocytes, or basophils. In contrast to earlier methods that rely on elaborate image preprocessing and manual feature engineering, the proposed approach requires no such preprocessing and feature handcrafting stage for classification. On top of this, the proposed method achieves the state-of-the-art results.

## Figures and Tables

**Table 1:** Comparison of the proposed approach with other works.

Method	Description of the approach
(Bikhet et al.) [28]	Image preprocessing, feature extraction, and classification
(Piuri and Scotti) [27]	Features extracted, feature selection, and classification
(Hiremath et al.) [29]	Advanced image processing, feature extraction, and classification
(Mathur et al.) [7]	Image processing, feature extraction, and classification
(Gautam et al.) [30]	Feature extraction and Naïve Bayes classifier
(Rawat et al.) [31]	Feature extraction and selection via PCA and classification
(Ours—GAN and DCNN)	No image processing. Automatic feature extraction. Data augmentation and classification via GAN and DNN, respectively

**Table 2:** Original LISC dataset details.

White blood cell type	Number of inst.
Neutrophils	50
Eosinophils	39
Lymphocytes	52
Monocytes	48
Basophils	53

**Table 3:** 10-fold cross-validation accuracy of the DNN models initialized using random weights.

Model	Original data (no aug.) (%)
VGG-16	90.6
VGG-19	91.8
ResNet-18	91.1
ResNet-50	92.7
DenseNet-121	93.9
DenseNet-169	94.4

**Table 4:** 10-fold cross-validation accuracy of the DNN models initialized using pretrained weights.

Model	Original data (no aug.) (%)
VGG-16	90.9
VGG-19	92.4
ResNet-18	91.5
ResNet-50	93.3
DenseNet-121	94.5
DenseNet-169	95.2

**Table 5:** 10-fold cross-validation accuracy of the DNN models initialized using random weights.

Model	Trans_aug1 (100 inst./class) (%)	Trans_aug2 (150 inst./class) (%)	Trans_aug3 (200 inst./class) (%)
VGG-16	91.5	92.1	92.9
VGG-19	92.3	92.8	93.4
ResNet-18	91.4	92.6	93.2
ResNet-50	93.5	94.0	94.7
DenseNet-121	94.4	94.8	95.4
DenseNet-169	94.9	95.4	95.8

**Table 6:** 10-fold cross-validation accuracy of the DNN models initialized using pretrained weights.

Model	Trans_aug1 (100 inst./class) (%)	Trans_aug2 (150 inst./class) (%)	Trans_aug3 (200 inst./class) (%)
VGG-16	91.4	91.8	92.5
VGG-19	92.9	93.6	94.4
ResNet-18	91.2	92.2	92.8
ResNet-50	94.1	94.8	95.5
DenseNet-121	95.2	95.7	96.4
DenseNet-169	95.8	96.4	96.9

**Table 7:** 10-fold cross-validation accuracy of the DNN models initialized using random weights.

Model	GAN_aug1 (100 inst./class) (%)	GAN_aug2 (150 inst./class) (%)	GAN_aug3 (200 inst./class) (%)
VGG-16	91.9	92.6	93.4
VGG-19	92.6	93.1	93.5
ResNet-18	92.7	94.0	94.6
ResNet-50	93.8	94.5	94.9
DenseNet-121	95.0	95.6	95.7
DenseNet-169	95.3	95.4	95.8

**Table 8:** 10-fold cross-validation accuracy of the DNN models initialized using pretrained weights.

Model	GAN_aug1 (100 inst./class) (%)	GAN_aug2 (150 inst./class) (%)	GAN_aug3 (200 inst./class) (%)
VGG-16	92.3	93.0	94.1
VGG-19	93.3	93.7	95.0
ResNet-18	92.9	93.7	94.2
ResNet-50	94.7	95.5	95.8
DenseNet-121	95.4	96.2	97.2
DenseNet-169	96.1	96.9	97.2

**Table 9:** 10-fold cross-validation accuracy of the DNN models initialized using random weights.

Model	Tran_aug1 + GAN_aug1 (200 inst./class) (%)	Tran_aug2 + GAN_aug2 (300 inst./class) (%)	Tran_aug3 + GAN_aug3 (400 inst./class) (%)
VGG-16	92.5	93.2	93.9
VGG-19	93.3	93.7	94.4
ResNet-18	93.2	94.5	95.1
ResNet-50	94.2	95.2	95.6
DenseNet-121	95.5	96.1	97.3
DenseNet-169	95.9	96.3	97.3

**Table 10:** 10-fold cross-validation accuracy of the DNN models initialized using pretrained weights.

Model	Tran_aug1 + GAN_aug1 (200 inst./class) (%)	Tran_aug2 + GAN_aug2 (300 inst./class) (%)	Tran_aug3 + GAN_aug3 (400 inst./class) (%)
VGG-16	94.3	94.9	95.7
VGG-19	94.8	95.4	95.9
ResNet-18	94.1	95.2	95.4
ResNet-50	95.8	96.7	97.4
DenseNet-121	96.3	97.4	98.3
DenseNet-169	96.9	98.1	98.8

**Table 11:** 10-fold cross-validation results comparison with other works.

Model	Train: test setting	Dataset	Acc. (%)
ResNet-50 (Tran_aug3 + GAN_aug3)	10-Fold CV	LISC	97.4
DenseNet-121 (Tran_aug3 + GAN_aug3)	10-Fold CV	LISC	98.3
DenseNet-169 (Tran_aug3 + GAN_aug3)	10-Fold CV	LISC	98.8
Linear discriminant analysis (LDA) [46]	10-Fold CV	Private	93.9
Neural network + PCA [47]	75%: 25%	Kanbilim	95.0
W-net [48]	10-Fold CV	Private	97.0
W-net [48]	10-Fold CV	LISC + private	96.0
Linear SVM [49]	10-Fold CV	CellaVision	85.0

## Data Availability

The datasets generated during and/or analysed during the current study are available from the corresponding author on reasonable request.
